# Conspiracy Theories

**DOI:** 10.1007/s12115-023-00816-1

**Published:** 2023-02-06

**Authors:** Quassim Cassam

**Affiliations:** University of Warwick, Coventry, CV4 7AL UK

**Keywords:** Anti-Semitism, Conspiracy theories, Conspiracy mentality, Generalism, Ideology, Particularism, Propaganda

## Abstract

Current thinking about conspiracy theories is dominated by epistemological and psychological approaches. The former see the study of conspiracy theories as a branch of epistemology and insist that each theory should be judged on its evidential merits. On this account, a conspiracy theory is an explanation of an event which cites a conspiracy as a salient cause. Psychological approaches explain belief in conspiracy theories by reference to individual personality traits and generic cognitive biases. Despite their popularity, both epistemological and psychological approaches are flawed. After identifying their flaws, a case is made for a different perspective which focuses on the *political* function of conspiracy theories. A conspiracy theory is not just an explanation of an event which cites a conspiracy as a salient cause. Conspiracy theories have a range of additional features which distinguish them from ordinary theories about conspiracies and make them unlikely to be true. The political approach sees many conspiracy theories as forms of political propaganda and is especially mindful of the role of conspiracy theories in promoting extremist ideologies.

## What Is a Conspiracy Theory?

In 1605, a small group of men, including Guy Fawkes, plotted to blow up the English Parliament at the Palace of Westminster. Fawkes was arrested on 5 November in the act of placing barrels of gunpowder in a vault beneath the House of Lords. The target of what would today be called a terrorist plot was the King. After his capture, Fawkes and his co-conspirators were tortured, tried, and executed. There are aspects of the so-called Gunpowder Plot that are still debated by historians. Some believe that it was, in modern parlance, a false flag operation by government agents. On the whole, however, the view that prevails and which is certainly believed by the British public is that the plot was genuine. The event is still commemorated annually in Britain by the lighting of bonfires on the 5th of November.^1^

The Gunpowder Plot was a classic conspiracy. A *conspiracy* consists in a small group of people working together in secret to do something illegal or harmful. A conspiracy *theory* has been defined as “*just* an explanation of an event which cites a conspiracy as a salient cause” (Dentith 2019: p. 102). To put it another way, conspiracy theories are simply “*theories* about *conspiracies*” (Dentith 2019: p. 94).^2^ On this definition, which will be referred to here as the *neutral* definition, the theory that Guy Fawkes and others plotted to blow up Parliament is a conspiracy theory. So is a more modern example: the theory that a small group of Al-Qaeda terrorists plotted the attacks on New York and Washington on what came to be known as 9/11. The sense in which the neutral definition is neutral is that it leaves open the question whether conspiracy theories are justified or unjustified, true or false.

Corresponding to the neutral definition of “conspiracy theory” is the definition of a conspiracy *theorist* as “someone who subscribes to a conspiracy theory” (Pigden 2007: p. 222). Far from implying any criticism of conspiracy theorists, this definition suggests that “every politically and historically literate person is a big-time conspiracy theorist, since every such person subscribes to a vast range of conspiracy theories” (Pigden 2007: p. 222). Only a person with no knowledge of politics or history could fail to subscribe to at least one conspiracy theory. Proponents of the neutral definition regard this as a welcome consequence of their approach. Opponents regard it as a *reductio*, and deny that the fact that the 9/11 Commission blamed the 9/11 attacks on an Al-Qaeda conspiracy made its members conspiracy theorists. The conspiracy theorists in this case are people who think that 9/11 was an inside job.^3^ One question is whether there is any rationale for describing the latter as conspiracy theorists but refusing to apply this label to the members of the 9/11 Commission.

A natural thought is that theories that are generally referred to as “conspiracy theories” are not just theories about conspiracies. In an important paper, Brian L. Keeley defines a conspiracy theory as a “proposed explanation of some historical event (or events) in terms of the significant causal agency of a relatively small group of persons – the conspirators – acting in secret” (Keeley 2006: p. 51). However, Keeley’s concern is not with conspiracy theories in this neutral sense. The theories that interest him have the following additional features: they (i) run “counter to some received, official, or ‘obvious’ account,” (ii) assume that “the true intentions behind the conspiracy are invariably nefarious,” (iii) “seek to tie together seemingly unrelated events,” (iv) assume that the truths behind the events they explain are well-guarded secrets, and (v) rely on “errant data” as their chief tool (Keeley 2006: pp. 51–52). Keeley calls conspiracy theories with these additional characteristics “unwarranted conspiracy theories” (UCTs). These are the theories that are usually labeled “conspiracy theories.” This label does not apply to the report of the 9/11 Commission, not least because its analysis of 9/11 did not run counter to the official view. It *was* the official view.

To mark the contrast between the neutral account and Keeley’s extended account of conspiracy theories, the latter might be described as *critical*. Another critical account says that what is special about conspiracy theories is that they are speculative (based on conjecture rather than solid evidence), contrarian (contrary to the received wisdom), baroque (extravagantly convoluted), amateurish (largely the work of amateurs rather than accredited specialists in the relevant subjects), and self-sealing (immune to counterevidence).^4^ On this approach, as on Keeley’s, neither the standard historical account of the Gunpowder Plot nor the official story about 9/11 is a conspiracy theory.^5^ The theory that 9/11 was an inside job or that climate change is a hoax is a conspiracy theory. Only people who believe these or other such unwarranted conspiracy theories are conspiracy theorists in the critical sense. Accordingly, it is not only possible but *easy* for a politically and historically literate person not to be a conspiracy theorist. Such a person should not and would not believe that 9/11 was an inside job or that climate change is a hoax.

Proponents of the neutral view seek to normalize and legitimize belief in conspiracy theories. Such *conspiracy apologists* see conspiracy theories as playing a positive role, that of raising questions about the conduct of those in positions of power.^6^ On this view, “conspiracy theories undermine the establishment by providing alternative facts, realities, and ways of knowing” (Uscinski 2019: p. 15). Conspiracy apologists also criticize what they call “conspiracy theory phobia.” A person has this phobia if “she rejects conspiracy theories without an appropriate evaluation of the *evidence*,” or if “her reaction toward particular conspiracy theories is mockery, contempt, hostility, or a straw-person characterization of the argument presented” (Räikkä and Basham 2019: p. 178). As well as highlighting the irrationality of these reactions, conspiracy apologists point out that theories that would once have been rejected as mere “conspiracy theories” have turned out to be correct and warranted in retrospect.

If a conspiracy theory is just an explanation of an event which cites conspiracy as a salient cause, then it is difficult to see why conspiracy theories as such should be dismissed as unwarranted. Some conspiracy theories are plausible, while others are not. On this view, the philosophy of conspiracy theories might be expected to identify “the epistemic features which make belief in conspiracy theories plausible or implausible” (Dentith 2019: p. 103). This broadly epistemological approach to conspiracy theories can be contrasted with two others. One is psychological, the other political. The main concern of psychological approaches is to explain in psychological terms why people *believe* conspiracy theories. This question is pressing if belief in conspiracy theories is assumed to be unwarranted. Political approaches, as defined here, are critical in their understanding of conspiracy theories. They focus on the ideological associations and underpinnings of conspiracy theories and on their political role.

The epistemological approach to conspiracy theories will be the focus of part 2. Part 3 will explore psychological approaches. Part 4 will focus on political approaches. The position taken here is that the philosophy of conspiracy theories should be sensitive to their political role and realistic about the extremist ideologies that they have been used to promote. On this view, philosophers who normalize and legitimize belief in conspiracy theories risk being apologists for repellent ideologies. Their understanding of conspiracy theories leads them to underestimate their harmfulness and exaggerate their benign nature as well as their benefits.^7^

## The Epistemology of Conspiracy Theories

It is easy and, in some ways, natural to think of conspiracy theories in epistemological terms. If conspiracy theories are truly just theories like any other, then there is no obvious a priori reason (a reason based on theoretical deduction rather than on empirical observation) to reject them in advance of detailed consideration of their evidential merits, unless they are self-contradictory. As with all theories that purport to explain a given event or type of event, there is the question whether the proposed explanation is a good one, and whether acceptance of a given conspiracy theory is epistemically justified. Thus, it would seem that the philosophy of conspiracy theories should concern itself with such familiar epistemological questions as: What makes an explanation of an event or action a good explanation? What makes belief in a theory epistemically justified? What counts as evidence for a theory? And so on. For example, one might suppose that a belief is justified to the extent that it has, and is based on, adequate evidential support and sound reasoning. As long as there is adequate evidential support for a conspiracy theory, belief in it is epistemically justified. Of course, there are conspiracy theories, the evidence for which is suspect or non-existent, but conspiracy theories are not unique in this regard. The evidence for a conspiracy theory can be untrustworthy, but so can the evidence *against* a conspiracy theory.

For M R. X. Dentith, these reflections support a particularist rather than a generalist view of conspiracy theories. Generalists claim that we have “justification for a general, *prima facie* suspicion of conspiracy theories. That is, given that conspiracy theories are either false, or…. suspect, we have grounds to treat the class of *conspiracy theories* dismissively” (2019: p. 94). In contrast, the particularist recommends that belief in conspiracy theories is assessed on a case-by-case basis. When appraising any conspiracy theory, “we have to assess it on its particular (read: evidential) merits, rather than treat it dismissively just because it has been labeled a conspiracy theory” (2019: p. 95). The neutral conception of conspiracy theories makes particularism unavoidable. If conspiracy theories can be warranted or unwarranted, who could object to the principle that every such theory should be assessed on its merits?

On a critical conception of conspiracy theories, matters are less straightforward than they are for the particularist. The critical conception does not stipulate that conspiracy theories are false, but it does imply that they are suspect and unlikely to be true. It is not the fact that they are contrary to the received wisdom or baroque that makes them suspect. Of greater significance is the fact that conspiracy theories in the critical sense are speculative, amateurish, and self-sealing. Why speculative? Because it is in the nature of conspiracy theories to rely on circumstantial rather than direct evidence. It would hardly be a conspiracy theory in the critical sense that 9/11 was an inside job if there is direct proof of this fact, in the form of documentary evidence or a confession by the alleged conspirators. It is the paucity or absence of direct evidence for the inside job theory that makes it necessary for 9/11 conspiracy theorists to connect the dots and rely on odd clues or anomalies that supposedly give the game away. Speculative conspiracy theories can still be epistemically justified but not as unproblematically as non-speculative theories.

An even greater problem for conspiracy theories on the critical conception is their amateurishness. Conspiracy apologists point to the uncovering of well-known conspiracies like Watergate but evidence of these conspiracies was discovered by well-placed individuals—journalists and whistleblowers—relying on solid evidence acquired in familiar ways. For example, the two journalists who uncovered the Watergate conspiracy, Bob Woodward and Carl Bernstein, had a source (“Deep Throat”) in the White House.^8^ In contrast, as Lewandowsky notes, a conspiracy theory “is discussed at length on the internet by people who are not *bona fide* journalists or government officials or investigative committees of regulators. They’re completely independent sources, individuals who self-nominate and put themselves forward as being in possession of the truth” (quoted in Allen 2020: p. 2). In other words, conspiracy theorists are mostly amateurs, and it is reasonable to regard their theories with suspicion.

Consider the case of Richard H. Popkin, the eminent historian of philosophy who was also the author of a book propounding a baroque conspiracy theory about the assassination of President Kennedy.^9^ Popkin was not in a position to know or find out that Lee Oswald, Kennedy’s assassin, was not working alone. Popkin was no investigative journalist and did not have the services of a Deep Throat. He was the archetypal amateur conspiracy theorist who lacked the technical expertise that would have been required for him to arrive at a genuinely well-informed conclusion about the assassination. Popkin was a professional philosopher, not a forensic pathologist or an expert in wound ballistics. Even without going into the details of his bizarre conspiracy theory, that theory is already suspect on account of his lack of relevant investigative expertise.^10^

The sense in which conspiracy theories are self-sealing is that “evidence that counters a theory is re-interpreted as originating from the conspiracy” (Lewandowsky & Cook 2020: p. 7).^11^ The absence of evidence for a conspiracy is re-interpreted by the conspiracy theorist as evidence of the conspirators’ ingenuity and success in covering their tracks. In the same way, contrary evidence is interpreted as a false trail laid by conspirators who want us to believe their version of events. By these means, conspiracy theories become immune to refutation by counterevidence, but theories that claim this kind of immunity are suspect, especially when their other characteristics are taken into account. To put it another way, a *prima facie* suspicion of conspiracy theories is justified, in line with generalism, even if we are also then prepared to assess these theories on a case-by-case basis, in accordance with particularism. It is possible to regard conspiracy theories as suspect but still be willing to examine the merits of individual theories, if only to establish whether one’s initial suspicions were justified in a given case. Generalism and particularism are, in this sense, compatible.

Underlying these concerns about a narrowly epistemological approach to conspiracy theories is a deeper point. This approach regards conspiracy theories as theories like any other, but this is hard to reconcile with the content of many actual conspiracy theories. Consider the *Protocols of the Elders of Zion*, a forgery dating from 1903 and supposedly describing a secret meeting at which a member of a group of Jewish elders outlines a fiendish plot for world domination. The story of the *Protocols* has been told by the historian Norman Cohn, who describes how they were used to justify the massacres of Jews during the Russian civil war and became an integral part of Nazi ideology. Quoted approvingly by Hitler, the *Protocols* helped to prepare the way for the Holocaust.^12^

The *Protocols* is hardly a “theory” to be considered on its merits. It is not *evidence* of a world Jewish conspiracy, and it would be bizarre to treat it as a serious attempt to explain an event by citing a conspiracy as a cause. There is no “event” to be explained, and the point of the *Protocols* is to scapegoat Jews with a view to inciting anti-Semitic violence. To think of it in any other way is to miss its fundamental point. It is not a serious candidate for truth or justification and has no potential merits that would warrant detailed consideration other than by historians of anti-Semitism. We have excellent grounds to treat the class of anti-Semitic or otherwise racist conspiracy theories dismissively. The appropriate reaction to them is a mixture of precisely the attitudes that supposedly mark one out as a conspiracy theory phobic: mockery, contempt, and hostility.

If it is objected that the *Protocols* is unrepresentative of modern conspiracy theories, it only needs to be pointed out that anti-Semitism remains a core theme of conspiracy theories to this day. A case in point is QAnon, a conspiracy theory which holds that a secret cabal of senior Democrats and Hollywood stars is involved in a plot to kidnap and abuse children with the aim of harvesting their blood. As Mia Bloom and Sophia Moskalenko point out, “QAnon is a modern day blood-libel that leverages many of the historical anti-Semitic stereotypes about Jews and the blood of babies” (2021: p. 30). As well as echoing the *Protocols*, QAnon folds in many other conspiracy theories and is increasingly gaining traction in America and elsewhere. A 2021 NPR/Ipsos poll found that 17% of Americans endorsed the central claims of the theory, with a further 37% claiming to be uncertain whether these claims are completely false.^13^

Even when conspiracy theories do not attempt to scapegoat minorities, they may have other disreputable objectives. For example, it is arguable that conspiracy theories about the 2012 massacre at Sandy Hook Elementary School in Connecticut were promoted by the alt-Right in America with a view to pre-empting calls for tighter gun control. More will be said about the politics of conspiracy theories in part 4. The key point for present purposes is that most conspiracy theories are political. To “epistemologize” them is to risk missing their point and overlooking their fundamentally ideological agendas.

Another way to bring out the limitations of the strictly epistemological approach is to take note of a phenomenon described by Russell Muirhead and Nancy Rosenblum. The discussion thus far has accepted that so-called conspiracy theories are *theories*, even if their political agendas make them theories of a distinctive sort. In a recent study, Muirhead and Rosenblum identify a new form of conspiracism: “conspiracy without the theory” (2019: p. 2). Classic conspiracism is “conspiracy *with* the theory” (2019: p. 2). It “tries to make sense of a disorderly and complicated world by insisting that powerful people control the course of events” (2019: p. 2). This sensemaking function of classic conspiracism explains why it appeals to so many: it renders intelligible events that would otherwise appear purely accidental or coincidental or inexplicable. Classic conspiracy theorists crave academic respectability and are in the habit of peppering their tracts with footnotes and citations, albeit of each other’s work.^14^ They accept the need to find evidence in support of their theories, and they seek to offer proofs. They are, at least to this extent, traditional in their methods.

The new conspiracism, one of whose most notable exponents is Donald Trump, is different:

There is no punctilious demand for proofs, no exhaustive amassing of evidence, no dots to form a pattern, no close examination of the operators plotting in the shadows. The new conspiracism dispenses with the burden of explanation. Instead, we have innuendo and verbal gesture: “A lot of people are saying…” Or we have bare assertion: “Rigged! . . . What validates the new conspiracism is not evidence but repetition . . . The new conspiracism – all accusation and no evidence – substitutes social validation for scientific validation: if *a lot of people are saying it*, to use Trump’s signature phrase, then it is true enough (Muirhead & Rosenblum 2019: p. 3).

The new conspiracism has what looks like a political agenda, albeit a purely negative one. It is “the pure face of negativity” (Muirhead & Rosenblum 2019: p. 7). It corrodes the legitimacy of democracy without suggesting an alternative. Its attacks on shared modes of understanding and assault on reality are exhausting and disorientating. Disorientation and delegitimation are its two main products.

Conspiracy apologists demand that conspiracy theories not be rejected without an appropriate evaluation of the evidence, but this demand makes no sense where no evidence is offered.^15^ New conspiracism is immune to counterevidence because it was never based on evidence in the first place. Conspiracy apologists see conspiracy theories as playing the positive role of raising questions about the conduct of those in positions of power, yet the leading new conspiracist was the President of the USA. Uscinski’s description of conspiracy theories as “seeking to undermine the establishment by providing alternative facts, realities, and ways of knowing” is closer to the truth about new conspiracism. However, there aren’t literally “alternative facts,” and the fact that a lot of people are saying that P does not make it true that P. New conspiracists revel in flouting the principle that one should only assert what one knows.

The narrowly epistemological approach to conspiracy theories makes little sense of conspiracism without the theory. It is one thing to judge conspiracy *theories* on their evidential merits, but what would it be to evaluate a one-word tweet on its evidential merits? Yet Muirhead and Rosenblum also claim that “the new conspiracism sheds political theory” (2019: p. 28). As they see things, “classic conspiracism is embedded in a more or less explicit ideology or political theory” (2019: p. 29). This is the central insight of the political approach to conspiracy theories, but this approach will struggle with the new conspiracism if “the new conspiracist mind-set is not ideological” (2019: p. 30). On this point, it is not clear that Muirhead and Rosenblum are correct. Much depends on how the notions of political theory and ideology are understood. We will return to these matters in part 4. Meanwhile, it is telling that Muirhead and Rosenblum refer to the *mind-set* of the new conspiracist. The idea that conspiracism is a mind-set is one that has been explored by psychologists. This would therefore be a suitable moment to turn to psychological approaches to conspiracy theories before returning to the role of ideology in conspiracy theorizing.

## The Psychology of Conspiracy Theories

In a widely discussed paper published in 1994, Ted Goertzel noted that “there is remarkably little psychological literature on belief in conspiracy theories” (1994: p. 735). That is not something that could reasonably be said today. Indeed, Goertzel’s paper significantly influenced subsequent investigations of the psychological causes of conspiracy theorizing. Partly as a result of the popularity of conspiracy theories about 9/11, the psychology of conspiracy theories is now an increasingly crowded field of research in social psychology.^16^ Research in this area, which has been further stimulated by conspiracy theories about the COVID-19 pandemic, has been dominated by two ideas. The first is that conspiracy theorizing is partly the result of what Rob Brotherton describes as “some of our brain’s quirks and foibles” (2015: p. 17). The second is that belief in conspiracy theories is a matter of personality, in that there are measurable individual differences in people’s willingness to accept conspiracy theories. People with a propensity to believe these theories are described as having a “conspiracy mentality” or as being “conspiracy minded.” To put it another way, they have a conspiracy *mind-set*. On this view, being conspiracy minded is a stable personality trait, and knowing that a person is conspiracy minded enables one to predict their response to a conspiracy theory they have not come across before.^17^

The quirks and foibles to which Brotherton refers include a range of cognitive biases such as intentionality bias (the tendency to assume things happen because they were intended rather than accidental), confirmation bias (the tendency to only pay attention to evidence that supports what one already believes while ignoring contrary evidence), and proportionality bias (the tendency to assume that the scale of an event’s cause must match the scale of the event itself). Intentionality and proportionality bias play a key role in generating conspiracy theories, while confirmation bias helps to sustain pre-existing theories. For example, when flight MH-370 disappeared in 2014 many explanations were put forward: the pilot and co-pilot deliberately crashed the plane, it was brought down by a missile strike, it was hijacked, it was the victim of a cyber-attack, and so on. What all these explanations have in common is the assumption that MH-370 vanished because somebody intended it. This is intentionality bias in action. The possibility that it was an accident is not taken seriously by the conspiracy minded.

Proportionality bias has been blamed for conspiracy theories about the assassination of President Kennedy in 1963. Conspiracy theorists find it hard to accept that somebody as insignificant as Oswald could have been responsible for the death of a President. Either he was not as insignificant as he seems, or other people were involved. And once proportionality bias has generated a conspiracy theory about the assassination, confirmation bias keeps it going in the face of a mountain of contrary evidence. Conspiracy theories about 9/11 have the same structure: conspiracy theorists cannot accept that a plot hatched in a ramshackle Al-Qaeda training camp in Afghanistan could possibly account for the total destruction of the World Trade Center and partial destruction of the Pentagon.^18^

Cognitive biases are universal but belief in conspiracy theories is not. How is it, then, that many people are not conspiracy theorists? Do their brains work differently from those of conspiracy theorists? This is not an inference that psychologists have been prepared to draw. They prefer to argue that “we are all natural-born conspiracy theorists” (Brotherton 2015: p. 17). This confuses the idea that we all believe some theories about conspiracies with the notion that we are all conspiracy theorists in the special sense in which people who believe that 9/11 was an inside job are conspiracy theorists. On a critical conception, many people (presumably including most readers of this article) are not inclined to believe conspiracy theories or to engage in conspiracy theorizing. The question this raises is whether such people are somehow immune to supposedly universal cognitive biases, or less susceptible to them, than conspiracy theorists. Cognitive bias approaches are not committed to regarding generic cognitive biases as sufficient for belief in conspiracy theories. Nevertheless, it remains true that in the rush to explain belief in conspiracy theories, psychological accounts are in danger of forgetting the increasingly neglected but widespread phenomenon of *dis*belief in conspiracy theories. The more natural belief in conspiracy theories is held to be, the more mysterious it seems that millions of people do not believe them.

The idea of conspiracy mindedness as a personality trait looks much more promising since there is no suggestion that everybody has this trait. Psychologists point to evidence that people who believe one conspiracy theory are more likely to believe other such theories, even totally unrelated theories. Goertzel argued that conspiracy beliefs make up what he dubbed a *monological* belief system. In a monological system, “each of the beliefs serves as evidence for each of the other beliefs,” and “the more conspiracies a monological thinker believes in, the more likely he or she is to believe in any new conspiracy theory which may be proposed” (1994: p. 740). Monological thinkers “do not search for factual evidence for their theories. Instead, they offer the same hackneyed explanation for every problem” (1994: p. 741). For example, a thinker who believes that 9/11 was an inside job may well be disposed to believe a conspiracy theory about the death of Princess Diana in a car crash. Yet there is no obvious connection between these events.

Goertzel’s idea was tested in a study by Wood et al. (2012).They found that people who subscribe to conspiracy theories are not only more likely to subscribe to other, *unrelated* conspiracy theories, they are also prepared to sign up to *contradictory* theories. People who believe that Princess Diana faked her own death (and so is still alive) also tend to believe that she was murdered (and so is dead). There is also the phenomenon of belief in completely fictitious conspiracy theories. Swami and his colleagues made up a conspiracy theory about Red Bull and tried it out on 169 women and 112 men from Austria. The theory included the claim that Red Bull contains substances that raise the desire for the drink and that the advertising slogan “Red Bull gives you wings” was chosen because in testing, rats who drank Red Bull literally grew wings. The study showed that the strongest predictor of belief in the fictitious conspiracy theory was belief in non-fictitious theories. This points to the existence of “a constellation of individual difference traits that are associated with conspiracist ideation” (Swami et al. 2011: p. 460).

Despite these findings, it would not be right to conclude that the conspiracy minded are wholly undiscriminating in their choice of conspiracy theories. In a study of American conspiracy theories, Uscinski and Parent (2014) found that liberals tend to be Truthers (to believe that President Bush ordered the 9/11 attacks) whereas conservatives tend to be Birthers (to believe that President Obama was not born in America). Faced by a conservative who is a Birther but not a Truther, one might ask why this person endorses one of these conspiracy theories but not the other. The notion that the individual concerned is conspiracy minded does not answer this question. It might explain their belief in conspiracy theories but not their belief in one theory rather than another. The explanation is political: the *particular* conspiracy theories (if any) to which people are drawn are the ones that accord with their broader political or ideological commitments.

This is also the lesson of belief in the *Protocols of the Elders of Zion*. Regardless of whether Hitler was conspiracy minded, it would be perverse to explain his belief in this theory without mentioning that fact that it was integral to his Nazi ideology. Hitler was receptive to anti-Semitic conspiracy theories because he was anti-Semitic. This is an essentially ideological explanation of his commitment to a particular conspiracy theory, though it leaves open the question why some people are susceptible to Nazi ideology. Even if being attracted to extremist ideologies is a personality trait, there is still the question why people are attracted to one extremist ideology in particular. There is considerable historical evidence of a link between conspiracy theories and extremism.^19^ It is possible to be a conspiracy theorist without being a political extremist, but most political extremists are conspiracy theorists. Political extremism is a risk factor for conspiracism, and vice-versa. It is a weakness of psychological approaches that they neglect the ideological foundations of conspiracy theorizing. A more overtly political perspective is needed.

## The Politics of Conspiracy Theories

One way that conspiracy theories can be political is by giving expression to a more or less specific political ideology. Thus, the *Protocols of the Elders of Zion* is an expression of the ideology of anti-Semitism. A different way for conspiracy theories to be political is for them to have a political agenda. Though conceptually distinct, these two political dimensions of conspiracy theories are related in practice: conspiracy theories promote the ideologies they express. Taken together, the expressive and promotional role of conspiracy theories constitute their *function*: the function of conspiracy theories is to express and promote an ideology.^20^ Some conspiracy theorists believe their own theories, while others do not.^21^ In other cases, it is hard to tell whether proponents of conspiracy theories believe their own theories, that is, whether they are sincere. However, conspiracy theories are more likely to fulfill their function if their consumers (as distinct from their producers) believe them.

Take the massacre of teachers and students at Sandy Hook Elementary School. The conspiracy theorists were quick to promote the idea that the episode was an elaborate hoax, a false flag operation by the Obama administration designed to bolster the case for tighter gun control. This theory expresses two core tenets of alt-right ideology—hostility to the federal government and opposition to gun control—while also promoting this ideology. If one were trying to design a conspiracy theory with the aim of fomenting hostility to government and pre-empting calls for tighter gun control, one could hardly do better than to promote the idea that nobody died at Sandy Hook. No great political insight is needed to discern the political foundations of Sandy Hook conspiracy theories.

One way to encapsulate this view of conspiracy theories is to think of them as forms of political propaganda.^22^ The *Protocols* is anti-Semitic propaganda and conspiracy theories about Sandy Hook are alt-right propaganda. Propaganda is the deliberate attempt to alter, reinforce, or otherwise affect a person’s political views and conduct by manipulating their emotions (Brennan 2017: p. 36). As Stanley (2015: p. 45) has noted, propaganda can be sincere. The Sandy Hook conspiracy theorist who sincerely believes the whole thing was a hoax will be no less effective at getting the anti-gun control message across than an insincere proponent of the same view. However, his sincerity does not mean what he says is not propaganda. Whatever his intentions, the function of his theory is to promote a political agenda by spreading seductive falsehoods. To talk about the “function” of conspiracy theories is to talk about what they are for, about the purpose they actually serve, rather than the intentions of their proponents.^23^ Some conspiracy theories are the work of what Sunstein and Vermeule call “conspiracy entrepreneurs” (2009: p. 212) who design their theories with a view to promoting a political cause. In these cases, conspiracy theories are witting rather than unwitting propaganda.

If conspiracy theories work by manipulating the emotions of their audience, they need to have emotional appeal. Their appeal has two sources. In the first place, conspiracy theories are *stories*, and the most seductive conspiracy theories have the emotional appeal of certain kinds of fiction. As Brotherton points out:

The best conspiracy theories have all the trappings of the classic underdog story. The enemy is formidable. From the Elders of Zion to the New World Order, from the weapons-industrial complex to Big Pharma, the names given to the conspirators often play up their overwhelming power and influence. Like every villain, however, the conspiracy has one fatal weakness; if only their schemes can be exposed to the light, the enemy becomes powerless (2015: pp. 149–50).

Apart from the David versus Goliath element of conspiracy theories, these theories are also seductive because they invest events with a meaning that they would otherwise lack. The death of Princess Diana in a car crash looks meaningless, but not if the crash was the result of a murder plot by the Royal Family. Conspiracy theories appeal to the quasi-religious impulse to look for a deeper meaning in prosaic events, to suppose that there *must* be more to them than meets the eye.^24^ The contrary view is that shit happens (see Mandik 2007).

If conspiracy theories are political propaganda, then conspiracy apologists risk being apologists for, or associating themselves with, the causes that conspiracy theories promote. Many of these are extremist or racist causes. On this issue, a source of valuable insights is the unjustly neglected work of Jovan Byford. One of Byford’s ideas is that conspiracism is what he calls a “tradition of explanation” (2011: p. 5). Different conspiracy theories sound alike because they are characterized by “a distinct thematic configuration, narrative structure and explanatory logic, as well as by the stubborn presence of a number of common motifs and tropes” (2011: p. 4). These include anti-Semitic motifs and tropes. Anti-Semitism is part of the historical DNA of conspiracy theories, many of which still explicitly or implicitly identify the Jews as the villains of their stories.^25^ Byford is correct in his observation that “for a substantial proportion of its history the conspiracy tradition was dominated by the idea of a Jewish plot to take over the world” (2011: p. 95). Even conspiracy theories that try to dissociate themselves from anti-Semitism “operate in an ideological space with a long antisemitic tradition” (2011: p. 100).

The idea that conspiracy theories operate in an ideological space, and that this space has an anti-Semitic tradition, contains an important lesson for conspiracy apologists and people who might be described as *conspiracy curious*, that is, receptive to conspiracy theories without being passionately committed. The lesson is that it is difficult to make excuses for conspiracy theories without also, at least implicitly, making excuses for the anti-Semitic tropes and motifs that have dominated the conspiracy tradition throughout its history. By the same token, flirting with conspiracy theories means flirting with the causes that they have promoted. Once the nature of these causes is understood, this ought not to be an alluring prospect. According to what might be called the *propaganda model* of conspiracy theories, it is difficult to be a conspiracy theorist or conspiracy apologist without coming into contact with the “antisemitic legacy of the conspiracy culture” (Byford 2011: p. 102).

This line of argument raises more questions than can be properly addressed here. The three most pressing are these:

1. How does the propaganda model of conspiracy theories account for the fact that some conspiracy theories have no political content?
2. What is the propaganda model’s take on new conspiracism? In particular, how does it respond to the suggestion that this form of conspiracism is not ideological?
3. Does the propaganda model rule out the possibility of conspiracy theories serving as propaganda for progressive causes? If so, is it right to rule this out?

On the first of these questions, a critic of the propaganda model might wonder whether, say, the theory that Elvis faked his own death is political, or what political cause is advanced by the theory that NASA faked the Apollo moon landings. Are these not counterexamples to the claim that the function of conspiracy theories is to advance a political cause?

Several responses to these questions are available to the propaganda model. The first is to allow that some conspiracy theories are apolitical while insisting that conspiracy theories are *generally* political, including many of the most well-known conspiracy theories. More to the point, supposedly apolitical conspiracy theories serve as a gateway to political theories. A person might start by taking an interest in theories with no obvious political content and then be drawn to theories that are overtly political. The point about conspiracy theories operating in an ideological space with its own traditions and history should also not be forgotten. Not even conspiracy theories about the disappearance of Elvis can avoid certain tropes and motifs that define the conspiracist tradition. To be drawn into this tradition is to risk being drawn into its darker recesses.

It should also be noted that the standard examples of supposedly apolitical conspiracy theories are less than compelling. The theory that Elvis faked his own disappearance is barely a conspiracy theory since there cannot be a conspiracy of one. On the other hand, conspiracy theories about the moon landings *are* political on a broad but still plausible conception of the “political.” These theories see the moon landing conspiracy as the work of elements of the so-called Deep State. The question raised by all such theories is: who were the conspirators, and why did they act as they did? The point at which the political content of moon landing conspiracy theories emerges is the point at which they try to answer these questions. In much the same way, anti-vaxxer conspiracy theories are political to the extent that they target so-called Big Pharma and thereby imply a more general criticism of big business capitalism.

The propaganda model’s take on new conspiracism is that while it might lack an explicit political theory, it is nevertheless embedded in a right-wing political tradition and has something that is recognizable as an ideology. An ideology is “a set of interrelated beliefs that provide a way for people to understand the world. Ideologies tell people what is important, who the good guys and bad guys are, what their goals are, and how those goals should be reached” (Uscinski & Parent p. 2014: 12). For the new conspiracists, the bad guys are the mainstream media and other members of the so-called elite. They have a way of understanding the world and are keen to promote their view of reality.

Even by Muirhead and Rosenblum’s own lights, the denial of an ideological basis to new conspiracism is far-fetched. They argue that:

The new conspiracism drains the sense that democratic government is legitimate without supplying any alternative standard. It operates at the level of citizens’ attitudes and emotions, insisting that the defining elements of political order are not worthy of support. This is delegitimation – a process of falling off from an earlier judgment that government has rightful authority (2019: p. 34).

Even if one were to accept this characterization, it offers little support for the idea that “the new conspiracist mind-set is not ideological” (2019: p. 30). Muirhead and Rosenblum’s account is an account of an ideology—an anti-democratic ideology. Whether their account is correct is another matter. The ideological motivating force of new conspiracism is not opposition to democracy as such but the belief that the *status quo* is not truly democratic and needs to be replaced by a different form of government that genuinely expresses the will of the people. As well as raising questions about the notion that new conspiracism is not ideological, this also calls into question the notion that new conspiracism is not, at least implicitly, a theory. To resolve this issue, greater clarity would be required about what constitutes a “theory” and what it is to “have” a theory.

Conspiracy apologists argue that conspiracy theories raise legitimate questions about the conduct of governments. They may argue, in addition, that the ideologies to which such theories give expression need not be regressive. Conspiracy theories about 9/11 were popular with liberals because they gave expression to their opposition to the Bush administration’s Iraq policy. Opposing the post-9/11 invasion of Iraq was politically progressive and had little to do with extremism. However, this attempt to rehabilitate conspiracy theories is flawed in a number of respects. As argued above, amateur conspiracy theorists are not best placed to hold governments to account. Their ill-grounded speculations about 9/11 were a nuisance but did not seriously inconvenience the Bush administration. It is possible to be critical of Bush’s Iraq policy without being a 9/11 conspiracy theorist, and critics of that policy who maintain that the President ordered the 9/11 attacks to justify the invasion of Iraq thereby dent their own credibility.^26^ Finally, it is worth noting that many 9/11 conspiracy theories are plainly anti-Semitic, including the theory that thousands of Jews employed by companies with offices in the World Trade Center did not go to work on 9/11, having been warned to stay away. In the world of conspiracy theories, even supposedly progressive theories, anti-Semitic tropes and motifs are always just around the corner. Progressives have plenty of legitimate reasons to be critical of the political *status quo* without resorting to conspiracy theorizing.

Given what is known about the history of conspiracy theories and the political agendas of some of the most influential conspiracy theories, it is remarkable that people who see themselves as politically progressive continue to expend intellectual energy on defending belief in conspiracy theories. They may say that they are not committed to defending every conspiracy theory and that they are entitled to reject a conspiracy theory on its evidential merits. However, even to talk about the evidential merits of a theory like QAnon is already to concede too much. The evidence for a theory is evidence of its *truth* but theories like QAnon are barely candidates for truth. Conspiracy theories which promote extremist, anti-Semitic ideologies need to be called out for doing so, and philosophers whose analyses even unwittingly give succor to such theories should know better.

## Footnotes

1 For more on the Gunpowder Plot, see Fraser (1996). Fraser distinguishes between “Pro-Plotter” and “No-Plotter” historians. The former believe in the plot’s existence. The latter “believe equally firmly that the Plot was a fabrication on the part of the government” (1996: p. xvi). Fraser accepts that there was a plot but dissents from the account of it given at the plotters’ trial. Pro-Plotters are not “conspiracy theorists” even though they believe there was a conspiracy. If the label “conspiracy theorist” applies to anyone, it is to No-Plotter historians. In Fraser’s words, “the events of 5 November 1605 have much in common with the killing of President Kennedy as a topic which is, in conspiratorial terms, eternally debatable” (1996: p. xvii).

2 Dentith’s paper appears in Uscinski (2019), which is the best collection of essays on conspiracy theories at the time of writing. Coady (2006) is another useful resource, and *Social Epistemology Review and Reply Collective* has also published some interesting material on conspiracy theories.

3 For a selection of conspiracy theories about 9/11, see Fetzer (2007) and Griffin (2004).

4 This is roughly the account of Conspiracy Theories given in Cassam (2019). There is one difference. I now describe such theories as baroque rather than esoteric. Based on the work of Richard Hofstadter, conspiracy theories in the problematic sense, and the people who believe them, might also be described as paranoid. See Hofstadter (2008). An example from history of a paranoid conspiracy theorist is Maximilian Robespierre. For an account of his conspiracist paranoia and its fatal consequences for thousands of his fellow citizens during the post-revolutionary Great Terror in France, see Tackett (2015).

5 What I am calling the standard historical account of the Gunpowder Plot is the “Pro-Plotter” account. See note 1 for an explanation of this terminology.

6 A number of academics who write about conspiracy theories are, in my terms, conspiracy apologists. There are also several academic philosophers who are not only conspiracy apologists but also conspiracy theorists. The two most notable are James Fetzer and David Ray Griffin. See Fetzer (2007) and Griffin (2004).

7 For an account of the many ways in which conspiracy theories are harmful, see chapter 3 of Cassam (2019).

8 The identity of Deep Throat is now known. He turned out to be Mark Felt, an FBI agent who died in 2005.

9 Popkin (2006).

10 For a much better account of the Kennedy assassination, see Posner (1993).

11 The “self-sealing” quality of conspiracy theories was identified and so named by Sunstein and Vermeule. For them, the self-sealing nature of conspiracy theories makes them “immune to challenge” (2009: p. 204).

12 Cohn (1967) is essential reading for anyone with an interest in the history and ideology of conspiracy theories.

13 These figures are quoted in Bloom and Moskalenko (2021): p. 3.

14 The craving for academic respectability and affinity for footnotes have both been remarked by Jovan Byford. See Byford (2011): pp. 89–90.

15 As Johnny Lyons pointed out to me in personal correspondence, there is an analogy here with rumors. There is no requirement to reject unsubstantiated rumors, however bizarre, only after an evaluation of the “evidence” on which they are based. Often there is no evidence, only a rumor whose absurdity warrants its rejection without further ado.

16 For an overview, see Wood and Douglas (2019).

17 See, for example, Swami et al. (2011).

18 The best account of the 9/11 plot is the one given in Wright (2007).

19 See Cassam (2022) for an analysis of extremism and the link between extremism and conspiracy theorizing.

20 See Cassam (2019) for an elaboration and defense of this view of conspiracy theories and an explanation of the idea of a function.

21 In a 2022 trial for defamation, Alex Jones, the main proponent of the Sandy Hook conspiracy theory, admitted that the massacre was “100% real”; *BBC News* (3 August, 2022): https://www.bbc.com/news/world-us-canada-62415376.

22 This is the propaganda model of conspiracy theories defended in Cassam 2019.

23 On this notion of the function of conspiracy theories, see Cassam 2019: pp. 10–11.

24 There is more on this aspect of the religious impulse in Crane (2017): p. 38.

25 Of course, there are also conspiracy theories that are not anti-Semitic, such as theories which target the Catholic Church. However, anti-Semitic theories have played a special role in the history of conspiracy theories and continue to be uniquely influential and pervasive today. See the above discussion of QAnon.

26 An eminent Bush critic and conspiracy theory skeptic is Noam Chomsky, who notes that conspiracy theories are a distraction from more serious matters. See Chomsky (2006).
